# Aged Garlic Extract Reduces IL-6: A Double-Blind Placebo-Controlled Trial in Females with a Low Risk of Cardiovascular Disease

**DOI:** 10.1155/2021/6636875

**Published:** 2021-03-31

**Authors:** Martiné Wlosinska, Ann-Christin Nilsson, Joanna Hlebowicz, Mohammed Fakhro, Malin Malmsjö, Sandra Lindstedt

**Affiliations:** ^1^Department of Cardiothoracic Surgery and Transplantation, Clinical Sciences, Lund University, Skåne University Hospital, Lund, Sweden; ^2^Department of Cardiology, Skåne University Hospital, Lund, Sweden; ^3^Department of Ophthalmology, Clinical Sciences, Lund University, Skåne University Hospital, Lund, Sweden

## Abstract

**Background:**

Chronic inflammation is a risk factor for cardiovascular disease. The aim of the study was to evaluate whether a daily supplementation of aged garlic extract (AGE) could reduce inflammation in females with low risk for cardiovascular disease. The study was conducted at a single center, as a parallel randomized placebo-controlled trial.

**Method:**

63 females with a Framingham risk score over 10 underwent cardiac computed tomography (CT) scan. Of those, patients with a coronary artery calcium (CAC) scores less than 5 (*n* = 31) met the inclusion criteria and were randomized, in a double-blind manner to an intake of placebo or AGE (2400 mg daily) for 1 year.

**Results:**

Main outcome measure was changes in inflammatory biomarkers, blood pressure, fastening blood glucose, and blood lipids. A total of 29 patients (14 in the AGE group and 15 in the placebo group) completed the study and were analyzed. Females treated with AGE showed lower levels of inflammatory marker IL-6 after 12 months of treatment compared to females receiving placebo (*p* < 0.05). The blood lipids had a trend towards a lowering effect in females treated with AGE; however, this trend was not significant.

**Conclusion:**

The present study concludes that AGE lowers IL-6 in females with a risk profile of cardiovascular disease. We could also conclude that risk prediction with cardiac CT  scan turned out to be superior in estimating the risk of cardiac disease compared to Framingham risk score. This trial is registered with NCT03860350.

## 1. Introduction

Cardiovascular diseases (CVDs) are among the leading causes of morbidity and mortality worldwide [1]. Atherosclerosis is one of the pathology processes behind CVD and can lead to ischemia of the heart, brain, or extremities, resulting in organ damage or infarction. The pathophysiology of atherosclerosis comprises a series of highly specific cellular and molecular responses that can be defined as an inflammatory disease and may be present throughout a person's lifetime [2].

A commonly used scoring system for CVD in primary care is the Framingham risk scoring, which is a gender-specific algorithm used to estimate the 10-year cardiovascular risk of an individual. It was first developed based on data obtained from the Framingham Heart Study, to estimate the 10-year risk of developing ischemic heart disease (IHD) [3]. However, the Framingham data, while thorough, are derived from many years ago with a potentially different USA population along in addition with a different diet and level of smoking, which may suggest different risk levels today. Recently, scoring the calcified atherosclerotic lesions in the coronary arteries, measured as coronary artery calcification (CAC) and its progression over time, has become a well-validated prognostic marker of IHD [4, 5].

Aged garlic extract (AGE) with the active ingredient S-allylcysteine (SAC) has been shown to have a positive effect on atherosclerosis, blood pressure, perfusion, blood lipids, and inflammation in cohorts with intermediate risk for CVD [6–17]. None of these studies have been carried out in a female cohort with a low risk of cardiovascular disease.

In the present study, females with a Framingham risk score ≥10 underwent a cardiac computed tomography (CT) scan. The females with no coronary artery calcifications, and thereby estimated to have a very low risk of CVD, were randomized to an intake of capsules of 2400 mg AGE daily (two capsules of 600 mg twice daily) or two placebo capsules twice daily for 12 months. Here, we describe in a randomized placebo-controlled trial of 29 females with a low risk of cardiovascular disease, the effect and tolerability of AGE as a primary, but also as an adjunct treatment on blood pressure, blood lipids, and inflammatory biomarkers. We also discuss the different risk prediction methods such as the Framingham risk score and coronary artery calcium (CAC) scores.

## 2. Methods

The study was designed as a double-blind placebo-controlled randomized study to determine whether AGE can influence inflammatory biomarkers (IL-6 and CRP), lipid profile, and blood pressure among females with a low cardiovascular risk profile. This study was conducted according to the guidelines laid down in the Declaration of Helsinki, and all procedures involving human patients were approved by the local ethical committee DNR 2016/745 (Lund, Sweden). All participants signed a written consent form before entering the study. The study protocol was registered at https://clinicaltrials.gov/ct2/show/NCT03860350?term=NCT03860350&rank=1 with ClinicalTrials.gov Identifier: NCT03860350. The study was monitored externally by Preventia AB, Sweden, https://www.preventia.se/en/startsida/. The study was conducted according to the CONSORT (Consolidated Standards of Reporting Trials) guidelines and statement [18]. The study was conducted in Sweden between October 2016 and October 2018.

### 2.1. Study Outcomes

The primary outcome was changes in inflammatory biomarkers (C-reactive protein (CRP) and interleukin-6 (Il-6)) after one year of placebo or AGE intake. Secondary outcome measurements were changes in blood pressure (diastolic and systolic), fasting blood glucose, and blood lipids (total cholesterol, high-density lipoprotein (HDL), low-density lipoprotein (LDL), apolipoprotein A and B, and triglycerides).

### 2.2. Inclusion Criteria

Asymptomatic patients between 40 and 75 years of age with a Framingham risk score ≥10 [3] and a cardiac computed tomography (cardiac CT) with calculated CAC score ≤5 were included. The subjects were required to be on stable concomitant medications for at least 4 months prior to randomization, and subjects with diabetes had to have an HbA1c <8.0 and stable HbA1c level (variation range within 0.5%) for 6 months.

### 2.3. Exclusion Criteria

(1) History of myocardial infarction, (2) symptoms of ischemic heart disease, (3) hypersensitivity to AGE therapy, (4) any unstable medical disorder, (5) bleeding disorder, (6) prior life-threatening arrhythmia, (7) resting hypotension (systolic <90 mmHg) or hypertension (resting blood pressure >170/110 mmHg), (8) heart failure, (9) history of malignancy within the last 5 years or evidence of active cancer, (10) serum creatinine >140 *µ*mol/L, (11) stroke, (12) triglycerides >4.0 mmol/L baseline visit, (13) diabetic subjects with HbA1c >8.0, and (14) drug abuse.

### 2.4. Randomization

A total of 63 females underwent cardiac CT scan. Of these, 31 patients met the inclusion criteria and were randomized in a double-blind manner, using numbered containers assigned to a computer-generated randomization chart by a study nurse. The patients were randomized to an intake of capsules with 2400 mg AGE daily (two capsules of 600 mg twice daily, Kyolic Reserve formula; Wakunaga of America Co., Ltd., *n* = 15) or two placebo capsules twice daily (starch capsules, *n* = 16) for 12 months. All patients receiving AGE supplement received the same dose. Study investigators, those assessing outcomes and patients, were blinded to treatment allocation.

### 2.5. Clinical Evaluation

Medical evaluation including medical history, cardiovascular risk factors, prescribed medications, smoking, and alcohol intake was performed at 0 and 12 months. In addition, blood pressure, body mass index, ECG measurements, and assessment of patients' compliance with medication were recorded. Blood pressure was measured after 10 minutes' rest in a comfortable supine position by an automatic blood pressure monitor (OMRON Automatic Blood Pressure Monitor Model M6 Comfort IT).

### 2.6. Blood Samples

Blood samples were collected at 0 and 12 months and analyzed using standard techniques. The following analyses were made: C-reactive protein (CRP), interleukin-6 (Il-6), fasting blood glucose, and blood lipids (total cholesterol, high-density lipoprotein (HDL), low-density lipoprotein (LDL), apolipoproteins A and B, and triglycerides).

### 2.7. CAC Measurements

Patients underwent cardiac CT with a 128-multidetector computed tomography scanner, SOMATOM Definition AS+ with Stellar detector by Siemens. Electrocardiographic triggering was performed at 70% of the R-R interval. The coronary arteries were imaged in sequential mode with 3.0 mm (Acq. 32 × 1.2 mm) axial slices. Measurement of Agatston Calcium score (CAC score) was performed with software, syngo.via, by Siemens. CAC score measurements were performed in noncontrast studies by an experienced reader blinded to the patient and clinical information. CAC was defined as a plaque of at least three contiguous pixels (area 1.02 mm^2^) with a density of >130 Hounsfield units. The lesion scores were calculated by multiplying the lesion area by a density factor derived from the maximal Hounsfield unit within this area, described as CAC score [19, 20]. The density factor was derived in the following manner: 1 for lesions with a peak attenuation of 130–199; 2 for lesions with a peak attenuation of 200–299; 3 for lesions with a peak attenuation of 300–399; and 4 for lesions with a peak attenuation of >400. Total calcium score was determined by summing individual lesion scores from each of the four main coronary arteries (left main coronary, left anterior descending coronary, left circumflex coronary, and right coronary arteries). Cardiac CT was performed prior to randomization. Only patients with a CAC score ≤5 were included. A CAC score ≤5 is considered a very low risk for coronary events [19, 20].

### 2.8. Statistical Analyses  and  Power Calculation of Study Cohort Size

A power calculation was made prior to the start of the study to calculate the adequate cohort size based on the research questions. The power calculation was based on prior studies evaluating the effect of garlic and supplements on blood pressure, cholesterol, and inflammatory biomarkers [11, 12, 21]. All continuous data are presented as a mean value ± SD or ± SEM, and all categorical data are reported as percentages or absolute numbers. Student's *t* tests and chi-square tests were used to assess differences between groups. Comparisons of all parameters between the active therapy and placebo were made with the Student's *t* test. All statistical analysis was performed using GraphPad Prism (Version 8, GraphPad Software, San Diego, USA). The level of significance was set to *p* < 0.05.

## 3. Results

Framingham risk score was used to predict the risk for CVD. A total of 63 females underwent cardiac CT  scan; of these, 31 females had a CAC score ≤5 indicating a low risk of coronary disease, whereas the remaining 31 females showed a significantly increased CAC score with a medium-to-high risk of coronary disease. The risk profiles for the excluded and the included groups are shown in Table 1.

**Figure 1:**
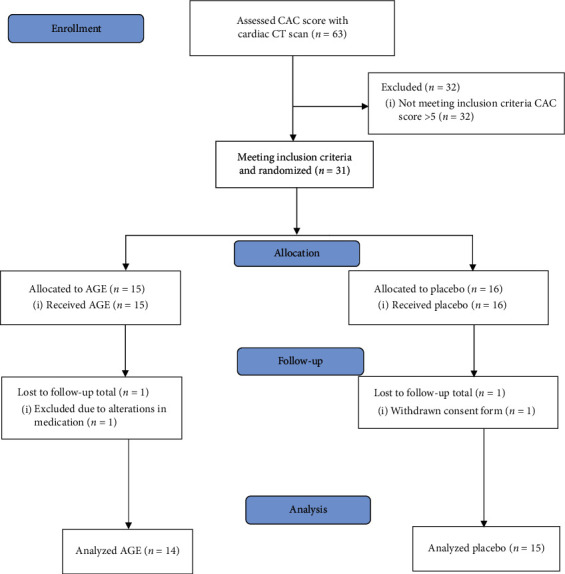
CONSORT statement (Consolidated Standards of Reporting Trials) flowchart showing demographics and baseline clinical information of the study cohort.

As stated, 63 females underwent cardiac CT  scan; of these a total of 31 patients met the inclusion criteria and were enrolled and randomized in the study, one participant withdrew consent, and a second altered her medication and was excluded, so consequently 29 patients, 14 in the AGE group and 15 in the placebo group, were analyzed (see CONSORT (Consolidated Standards of Reporting Trials) outlined in Figure 1). No patient in the study had any adverse reaction to the active therapy that required removal from the study.

At baseline, there were no significant differences in cardiovascular risk factors calculated using the Framingham risk score. The majority of the patients in the study were taking medications for hypertension and had family history of CVD when they entered the study. There was a significant difference in hypercholesterolaemia between the two groups when entering the study. Patient demographics are shown in Table 2.

Baseline characteristics and absolute values are presented in Table 3. There was no significant difference between the AGE group and the placebo group at baseline measurements at 0 months in BMI, blood pressure, blood lipids, or in inflammatory markers.

The mean annual changes in percent (%) for BMI, blood pressure, lipids, homocysteine, and CRP are shown in Table 4. Note a trend towards a lipid-lowering effect in the AGE group; however, this was not significant.

**Figure 2:**
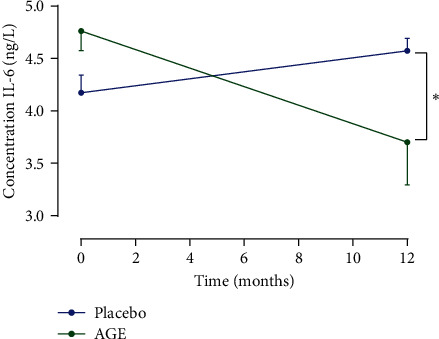
Interleukin-6 concentration at 0 and 12 months of either AGE or placebo treatment. Data are presented as mean ± SEM. The level of significance was set to *p* < 0.05.

IL-6 was measured at 0 and 12 months of either AGE or placebo treatment. At baseline, at 0 months of treatment of either AGE or placebo, the IL-6 concentration was 4.762 ± 0.701 ng/L in the AGE group and 4.173 ± 0.653 ng/L in the placebo group (*p* > 0.05). After 12 months of treatment, the IL-6 concentration was 3.754 ± 0.493 ng/L in the AGE group and 4.573 ± 0.461 ng/L in the placebo group (*p* > 0.05). The differences between the two groups were calculated as mean annular percent change (*p* < 0.05). (Figure 2).

No adverse events or side effects were reported from any of the participants.

## 4. Discussion

The present study concluded that 12 months of AGE treatment had a lowering effect on the inflammatory biomarker IL-6. AGE has in prior clinical and preclinical studies shown a beneficial effect on inflammation [22, 23], with a significant lowering effect on IL-6; however, this is the first time it has been shown to have a lowering effect in a female cohort with a low risk of cardiovascular disease.

AGE has immunomodulatory effects, among other things, through its application of antioxidants. S-1-propenylcysteine (S1PC) and SAC are the two predominant sulfur-containing amino acids present in AGE [24]. S1PC modulates antioxidant gene expression via the NO (nitric oxide)/heme (heme oxygenase-1)/BACH1 (BTB domain and CNC homolog 1) signaling pathway by the property of downregulating BACH1 in a NO-dependent manner and at the same time enhancing the expression of antioxidant genes reciprocally regulated by nuclear factor erythroid 2-related factor 2 (NRF2) and BACH1 [25].

S1PC also modulates the immune response by inducing autophagy, a key event in cellular recycling processes due to its involvement in the intracellular degradation of proteins [26]. S1PC degrades the adaptor protein myeloid differentiation response protein 88 (MyD88) of downstream of Toll-like receptor (TLR) by activating autophagy [26]. The degradation of MyD88 inhibits the TLR signaling pathway, including the phosphorylation of IL-1 receptor associated kinase 4 (IRAK4) and nuclear factor (NF)-kappa*β* p65, that leads to the inhibition of IL-6 production and C-C motif chemokine ligand 2 (Ccl2) mRNA expression [26].

AGE ameliorates atherosclerosis and type 2 diabetes through the suppression of inflammation. AGE modulates the inflammatory response by enhancing the phosphorylation of AMP-activated protein kinase (AMPK) [23]. AMPK plays an important role in regulating the inflammatory response through the inhibition of the TLR signaling pathway. Therefore, AGE may prove to be useful for the prevention and improvement of various diseases associated with chronic inflammation [23].

IL-6 is known to be involved in the inflammatory processes of atherosclerosis [27], potentially by its involvement in the acute phase responses of tissue injury and in chronic inflammation where an elevation of the Il-6 level is seen [28]. Il-6 has been shown to reduce insulin sensitivity in hepatocytes and has also been shown to be an independent predictor of type 2 diabetes mellitus and its associated cardiovascular events [29]. Furthermore, elevated levels of IL-6 are also seen during viral and bacterial infections, physical exercise, and oxidative stress [30].

AGE and other natural products with garlic (*Allium sativum*) have been shown to have a positive impact on vascular endothelial and platelet function, both playing a pivotal role in the etiology of arteriosclerosis and cardiovascular disease [7, 31–35]. IL-6 also stimulates low-grade inflammatory processes involved in the pathogenesis causing type 2 diabetes. Mediators of inflammation such as IL-6 have been suggested to be involved in these events. IL-6 has, in addition to its immune regulatory actions, been proposed to affect glucose homeostasis and metabolism both directly and indirectly by its action on skeletal muscle cells, adipocytes, hepatocytes, pancreatic beta-cells, and neuroendocrine cells [36]. After evaluating the annual percent change in CRP, an almost 28% lowering effect was noted in the AGE cohort; however, the change was not significant.

Assessment of the mean annual change in blood lipids between the two cohorts revealed that the AGE cohort experienced a lowering effect on triglycerides, and the placebo group showed a more than 50% increase in triglycerides; however, the change was not significant. The same pattern was seen when LDL was evaluated, with a lowering effect in the AGE cohort and an increasing effect in the placebo cohort, but again these changes were not significant. Several previous placebo-controlled studies have shown that AGE has a significant lipid-lowering effect [6, 11, 37].

All females in both cohorts had very well-regulated blood pressure when entering the study. All women had blood pressure that was within the optimal value for blood pressure control, and the majority of the females in both groups were on antihypertensive treatment when entering the study and when finishing the study. Previous studies have shown that AGE has antihypertensive properties, which we could not demonstrate in the current study. Possibly the cohorts' blood pressure was so well regulated and well-adjusted at the start of the study that this was the reason that a blood pressure lowering effect was not seen, but it could also possibly be because the cohorts were too small for such a change to be observed [11, 21, 38–40]. Calculation of the degree of calcification of the coronary arteries using cardiac CT scan and CAC score has been shown to predict coronary events beyond the Framingham risk score risk factors [41]. In the present study, 63 females with a Framingham risk score ≥10 underwent cardiac CT scan; of these, 31 females had an CAC score ≤5 indicating a low risk of cardiac events, whereas the remaining 31 females showed a significantly increased CAC score with a medium-to-high risk of cardiac disease. Comparing the two groups, two major differences between the groups were seen regarding if the patients were active smokers or had hyperlipidemia, where of the females with low risk, almost none had hyperlipidemia and not a single one was an active smoker. The difference was not significant, but this could be just like the unobserved antihypertensive effect because the cohorts were too small for such a change to be observed. The females in both cohorts were under antihypertensive treatment.

## 5. Limitations

The Framingham risk score was used in the present study and has been validated as a useful tool in the estimation of the 10-year risk of IHD mainly in primary care [3]. However, events may still occur among those predicted to be at low risk of IHD and some individuals might even be overestimated [42, 43]. With regard to limitations of the Framingham risk score for risk prediction, much effort has been directed towards improving identification of individuals at risk of coronary events. The serum levels of S-allylcysteine, to ensure intake, was not measured in the AGE supplement group. However, the patients were taken into clinical check-ups every 3 months and every month the study nurse was in contact with the patient to ensure intake of placebo or AGE supplement capsules.

## 6. Conclusions and Future Perspective

The present study concluded that AGE lowers IL-6 in females with a low risk profile of cardiovascular disease, as estimated with cardiac CT scan. We were also able to conclude that risk prediction with cardiac CT  scan in females was superior in estimating the risk of coronary disease than the Framingham risk score. As a future perspective, it would be interesting to compare treatment with SAC or S1PC alone, to understand its mechanism of action in clinical applications.

## Figures and Tables

**Table 1:** Patients' risk profiles (one patient withdrew consent before background information was confirmed and one patient in each group did not answer the questions on smoking status and family history).

Variable	Included (*n* = 31)	Excluded (*n* = 32)	*p*
	%	%	
Age (years) (SD)	62.55 (SD 4.8)	66.13 (SD 5.75)	0.009
Gender (female)	31	32	1.0
Hypertension	73	78.1	0.67
Hypercholesterolaemia	43	65.6	0.08
Diabetes mellitus	20.7	18.8	0.90
Current smoker	0	10	0.08
Family history of CVD	90	97	0.297
Framingham risk score (SD)	15 (SD 3)	19 (SD 6)	0.41
CAC score (SD)	0.44 (SD 1.33)	138.07 (SD 156.87)	<0.001

**Table 2:** Patients' demographics (one patient in the AGE group did not report smoking status).

Variable	AGE (*n* = 14)	Placebo (*n* = 15)	*p*
	%	%	
Age (years) (SD)	62.6 (SD 5.2)	62.8 (SD 4.9)	0.9
Gender, female	14	15	1.0
Hypertension	79	67	0.49
Hypercholesterolaemia	21	60	0.035
Diabetes mellitus	21	20	0.93
Current smoker	0	0	1.0
Family history of CVD	93	100	0.34
Framingham risk score (SD)	15 (SD 3)	14 (SD 2)	0.83

**Table 3:** Baseline characteristics and absolute values at 0 months.

Variable	AGE		Placebo		*p*
	*n* = 14	SD	*n* = 15	SD	
BMI	28.3	(5)	27.8	(5.1)	0.80
Systolic BP (mmHg)	145.6	(15)	146.9	(17.4)	0.59
Diastolic BP (mmHg)	83.1	(15.6)	87.8	(10.4)	0.68
Triglycerides (mmol/L)	1.2	(0.6)	2.2	(3.4)	0.22
Cholesterol (mmol/L)	6.0	(1.3)	6.0	(0.9)	0.88
HDL (mmol/L)	1.7	(0.6)	1.9	(0.4)	0.66
LDL (mmol/L)	4.1	(1.4)	3.7	(1.1)	0.60
CRP (mg/L)	3.0	(2.7)	1.6	(2.1)	0.93
ApoB/ApoA1	0.7	(0.3)	0.6	(0.2)	0.34
Homocysteine *µ*mol/L	12.6	(3.2)	12.1	(2.7)	0.55
Glucose (mmol/L)	6.2	(1)	6.3	(1.2)	0.76
Interleukin-6 (ng/L)	4.8	(2.7)	4.2	(2.4)	0.537

SD, standard deviation; Apo B/ApoA1, apolipoprotein B (mmol/L)/apolipoprotein A1 (mmol/L); BMI, body mass index (kg/m^2^).

**Table 4:** Mean annual changes.

Variable	AGE		Placebo		*p*
	*n* = 14	SD	*n* = 15	SD	
BMI	0.2	(1.7)	0.1	(2.4)	0.88
Systolic BP (mmol/L)	0.9	(12.8)	3.0	(13.9)	0.40
Diastolic BP (mmol/L)	7.9	(34.5)	0.0	(10.1)	0.44
Triglycerides (mmol/L)	−0.3	(39.3)	52.2	(236.7)	0.94
Cholesterol (mmol/L)	−3.9	(12.3)	−3.6	(11.8)	0.94
HDL (mmol/L)	1.1	(13.1)	1.4	(12.8)	0.38
LDL (mmol/L)	−1.6	(15.1)	4.6	(20.7)	0.15
CRP (mg/L)	−27.8	(39.4)	11.3	(65.2)	0.69
ApoB/ApoA1	0.5	(11.6)	2.3	(13.4)	0.08
Homocysteine (*µ*mol/L)	1.1	(15.7)	12.9	(18.3)	0.62
Glucose (mmol/L)	1.9	(12.7)	−0.4	(10.2)	0.36

SD, standard deviation; Apo B/ApoA1, apolipoprotein B (mmol/L)/apolipoprotein A1 (mmol/L); BMI, body mass index (kg/m^2^).

## Data Availability

The datasets used and/or analyzed during the current study are available from the corresponding author on reasonable request.

## Abbreviations

AGE: Aged garlic extract
CAC: Coronary artery calcification
CRP: C-reactive protein
CT: Computed tomography
CVD: Cardiovascular disease
HbA1c: Glycated hemoglobin
HDL: High-density lipoprotein
IHD: Ischemic heart disease
Il-6: Interleukin-6
LDL: Low-density lipoprotein
SAC: S-allylcysteine
S1PC: S-1-propenylcysteine
NO: Nitric oxide
BACH1: BTB domain and CNC homolog 1
NERF2: Nuclear factor erythroid 2-related factor 2
MyD88: Myeloid differentiation response protein 88
TLR: Toll-like receptor
IL-1: Interleukin-1
IRAK4: Receptor associated kinase 4
NF: Nuclear factor
Ccl2: C-C motif chemokine ligand 2
AMPK: AMP-activated protein kinase.
